# Conjoined twins—thoraco-omphalopagus (type A)

**DOI:** 10.1259/bjrcr.20150016

**Published:** 2015-10-22

**Authors:** Omar Muayad Sultan, Ahmed Said Tawfeek

**Affiliations:** ^1^ College of Medicine, Tikrit University, Tikrit, Iraq; ^2^ Nasser Institute Hospital, Cairo, Egypt; ^3^ Al Noor Hospital, Madinat Zayed City, UAE

## Abstract

A case of conjoined twins discovered by routine transabdominal ultrasound examination at 16 weeks gestation in a 19-year-old multigravid female. They were joined at the chest and the abdomen and had one functional heart. The pregnancy was terminated by caesarean section at 19 weeks gestation with approval from the family.

## Summary

A case of conjoined twins discovered by routine transabdominal ultrasound examination at 16 weeks gestation in a 19-year-old multigravid female. They were joined at the chest and the abdomen and had one functional heart. The pregnancy was terminated by caesarean section (CS) at 19 weeks gestation with approval from the family.

## Case report

### Clinical presentation

A 19-year-old multigravid female was visiting our private clinic for a routine antenatal transabdominal ultrasound check at 16 weeks gestation. Two foetuses joined at the chest and abdomen were identified by two-dimensional ultrasound, and further confirmation was made by three- and four-dimensional ultrasound. There was blood incompatibility between the parents (the husband was A+ whereas the mother was A–). Anti-D was previously administered after delivery of her first child, a single viable healthy male baby. She had no history of abortion or miscarriage. Her height was 165 cm, weight 63 kg, looked normal, was neither anaemic nor hypertensive and had no history of chronic diseases.

The last menstrual date of this twin pregnancy was unknown. The mother did experience normal foetal movements. She had no vaginal discharge or hyperemesis gravidarum. Her abdomen was slightly large for the date of pregnancy. There was no family history of twins.

### Investigations and imaging findings

**Figure 1.**
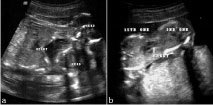
Gray scale transabdominal ultrasound. (a) Longitudinal and (b) transverse views show the fusion site with single central heart.

**Figure 2.**
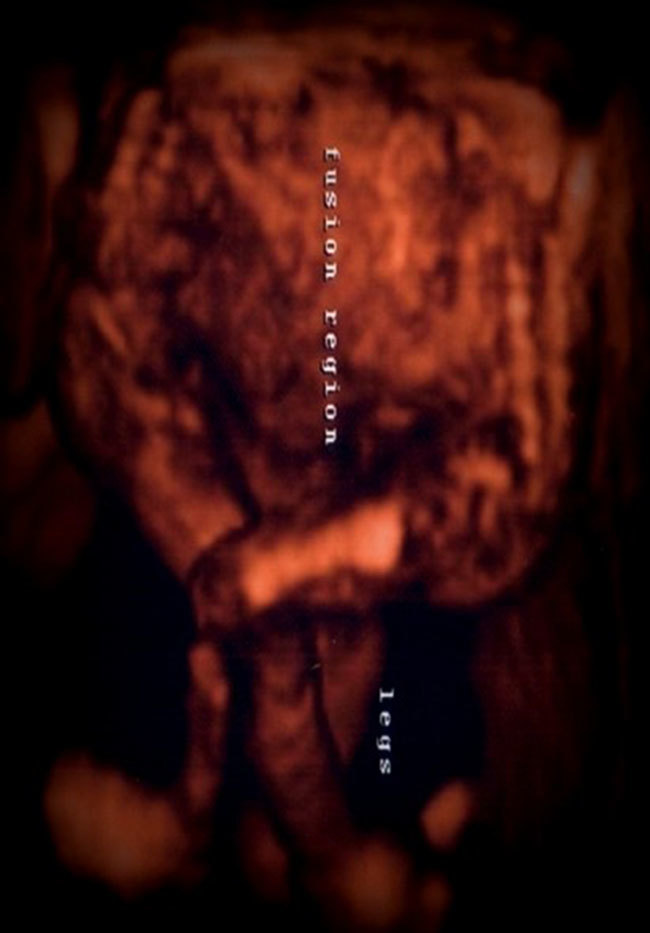
Three-dimensional ultrasound image shows two fused foetuses with free lower limbs.

**Figure 3.**
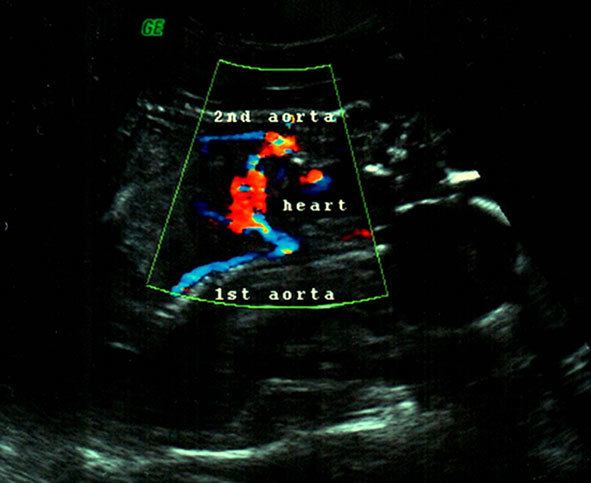
Transabdominal colour Doppler ultrasound shows single central heart with two aortic arches and two descending aortas.

A Voluson 730 ultrasound machine from GE Healthcare (Waukesha, WI) was used. The transabdominal ultrasound was performed and two conjoined female foetuses were detected; each foetus was with a single head and a pair of arms and legs. The twins were joined at the lower chest and the upper abdomen. Only one functional foetal heart was observed centrally between both the foetuses with slight tendency towards one of the foetuses (Figures 1 and 2). Single anterior placenta was seen with the umbilical cord and a normal amount of liquor. The colour Doppler study showed two aortic arches emerging from the single functional heart (Figure 2). On the basis of these findings, a diagnosis of dicephalic thoraco-omphalopagus conjoined twins was made (Figure 3).

### Outcome

**Figure 4.**
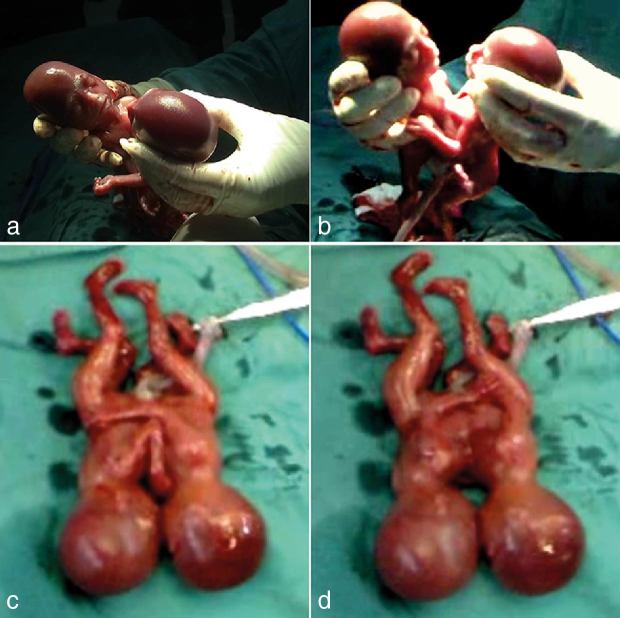
Photographic images (a–d) show the thoraco-omphalopagus conjoined twins immediately after delivery.

The parents were informed of the malformation and the likely outcome if the twins survived after delivery. They decided to terminate the pregnancy and refused further evaluation and investigation. A CS was performed at the request of the parents in the hospital and the delivery of viable conjoined twins aged 19 weeks was achieved without complication (Figure 4 and Supplementary Video). The conjoined twins died a few minutes after delivery.

## Discussion

Conjoined twins are popularly known as Siamese twins. The original Siamese twins were born in Siam (now Thailand) in 1811. They were males and lived for about 62 years. They moved to the USA where they worked in circuses, then became subjects of scientific research and finally farmers. They married two sisters in 1843 and fathered 22 children.^1,2^

Conjoined twinning is a random event, unrelated to hereditary factors.^3^ No maternal age effect has been found.^4^ They are considered rare forms of twin gestations. Conjoined twinning results from late twinning of a single zygote. The incidence rate is about 1 in every 200 identical twins. The estimated incidence of this phenomenon is between 1 in 50,000 to 100,000 live births.^5,6^ It is more frequently found among females with a ratio of 3 : 1.^1,7^ Conjoined twins share a single common chorion, placenta and amniotic sac. However, these features are not unique for conjoined twins, as some monozygotic twins who are non-conjoined share the same structures.^7,8^ Similar to all monozygotic twins, all conjoined twins also have the same sex.^7^

The medical term used to describe conjoined twins ends with the suffix “pagus” from the Greek word “fixed”. Five types of conjoined twins have been described:

1. Thoracopagus (joined at thorax).
2. Omphalopagus (joined at the anterior abdominal wall).
3. Craniopagus (joined at the cranium).
4. Syncephalus (joined twins with one head).
5. Ischiopagus (joined at the buttocks).^3^

The most common form of conjoined twins is fusion of the anterior thorax and/or abdomen (referred to as thoracopagus, omphalopagus and thoraco-omphalopagus—as in our case), which altogether constitutes 70% of conjoined twins.^8^ Increased nuchal fold thickness has been documented in multiple cases of conjoined twins. It is mainly associated with thoracopagus conjoined twins.^9^

The cause of conjoined twins is unknown. Two theories have been postulated to explain the origin of this phenomenon:

1. Fission theory is the traditional one in which the fertilized egg is incompletely split, causing delayed separation of the embryonic mass after day 12 of fertilization.
2. Fusion theory, in which the fertilized egg is completely separated, but the stem cells fuse with like-stem cells in the other twin, leading to fusion of both twins together.^6^–^8,10,11^

Conjoined twins are reported as early as the 10th week of gestation.^10^ When the diagnosis of the conjoined twins is made, the type and severity of the abnormality should be assessed with two- and three-dimensional ultrasound, CT scan or MRI.^4,9^ They are generally incompatible with life. 65% of cases were stillborn, and of those that were born alive, 35% died within the first 24 h. Only 25% survived to an age where surgical separation could be performed.^1^

The surgical management of the conjoined twins recognizes three categories:^10^

- Category I: no surgical intervention should be considered when the cardiac fusion is such that it is not possible to construct one single functioning heart.^10^
- Category II: emergency separation in case of:
  - death of one of the twins
  - one of the twins is dying and threatening the life of the other
  - presence of an anomaly that is incompatible with life if left untreated.^10^
- Category III: planned separation when the infants’ condition is deemed stable to perform the necessary imaging investigations that allow precise mapping of the separation.^10^

The ideal timing for separation would be around 3 months of age when the physiological condition and pliability of tissues are optimal. A tertiary referral centre with an experienced medical team of surgeons, anaesthetists and intensivists is required in case of surgical intervention. Furthermore, success depends on previous experience.^10^

The first successful operation to separate conjoined twins was performed by Professor Doyen in 1902 in Paris for the separation of the Radica–Doodica sisters, one of whom had developed abdominal tuberculosis and died but the other survived.^12^

The likelihood of a successful separation is dependent on good prenatal imaging with ultrasound and MRI.^13^ The major role of imaging is to analyze the extent to which the organs are shared so that a reasonable assessment of surgical separability can be made. Sharing of heart or brain virtually excludes separability.^14^

Surgical separation depends on the conjoining site and the organs that are shared. It can range from a relatively simple to a very complicated operation, and most of them are risky and life-threatening.^7^ Separation is unlikely to succeed if the hearts are united. However, if the hearts are separate, the success of the surgery depends on the status of the other organs.^15^ CS is indicated when surgical separation and viability is possible to minimize complications to the foetus and the mother.^4^

When severe forms are diagnosed prior to 24 weeks gestation, termination via vaginal delivery should be considered.^1^ In our case, the conjoined twins were joined at the chest and the abdomen forming a thoraco-omphalopagus, with single functional heart and two aortas. They were classified as Category I, where no operative management is possible. The diagnosis was made in the second trimester (16 weeks gestation) and the family chose termination. The pregnancy was terminated by CS; however, there was no indication for CS and a normal vaginal delivery should have been attempted. CS may have been attempted owing to a lack of clear local guidelines and experience in dealing with a condition of this rarity.

## Learning points

1. Early correct prenatal diagnosis is essential for better obstetric management and treatment planning.
2. Ultrasound is the modality of choice for prenatal detection of conjoined twins while MRI is the modality of choice for better characterization.
3. Termination of pregnancy, especially at a late stage, is fraught with problems.
4. When severe forms of conjoined twinning are diagnosed prior to 24 weeks, termination via vaginal delivery should be considered.
5. CS is indicated when surgical separation and viability is possible to minimize complications to the foetus and the mother.
6. The risk from termination to the mother is higher the more advanced the pregnancy is.
7. There are also social and legal issues with late termination, influenced by cultural and religious beliefs. This stresses the need for early and accurate diagnosis to enable a correct management plan, thus mitigating those potential complications.
